# Stroke‐Induced Renal Dysfunction: Underlying Mechanisms and Challenges of the Brain–Kidney Axis

**DOI:** 10.1111/cns.70114

**Published:** 2024-11-12

**Authors:** Xi Chen, Dong‐Xiao Yang, Heng Zhao, Hong‐Fei Zhang, Pu Hong

**Affiliations:** ^1^ Department of Anesthesiology Zhujiang Hospital of Southern Medical University Guangzhou China; ^2^ College of Anesthesiology Southern Medical University Guangzhou China; ^3^ Beijing Institute of Brain Disorders, Laboratory of Brain Disorders, Ministry of Science and Technology, Joint Innovation Center for Brain Disorders Capital Medical University Beijing China

**Keywords:** AKI, brain–kidney interaction, CKD, renal dysfunction, stroke

## Abstract

Stroke, a major neurological disorder and a leading cause of disability and death, often inflicts damage upon other organs, particularly the kidneys. While chronic kidney disease (CKD) has long been established as a significant risk factor for cerebrovascular disease, stroke can induce renal dysfunction, manifesting as acute kidney injury (AKI) or CKD. Mounting clinical and basic research evidence supports the existence of a bidirectional brain‐kidney crosstalk following stroke, implicating specific mechanisms and pathways in stroke‐related renal dysfunction. This review analyzes pertinent experimental studies, elucidating the underlying mechanisms of this cerebro‐renal interaction following stroke. Additionally, we summarize the current landscape of clinical research investigating brain‐kidney interplay and discuss potential challenges in the future. By enhancing our understanding of the scientific underpinnings of brain‐kidney crosstalk, this review paves the way for improved treatment strategies and outcomes for stroke patients. Recognizing the intricate interplay between the brain and kidneys after stroke holds profound clinical implications.

## Graphical abstract

After a stroke, multiple pathways in the neurohumoral system are activated. At the same time, the blood–brain barrier is compromised, and pro‐inflammatory substances are released from the brain into the bloodstream, consequently activating the peripheral inflammatory immune response. As an acute injury, stroke triggers renal hemodynamic disturbances and an acute ischemic state in the kidney, thereby promoting the development of AKI or CKD.

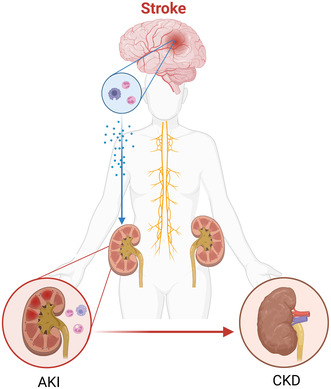

**Abbreviations**
ACR: albumin–creatinine ratio
ACTH: adrenocorticotropic hormone
ADH: antidiuretic hormone
AIS: acute ischemic stroke
AKI: acute kidney injury
BBB: blood–brain barrier
BP: blood pressure
CBF: cerebral blood flow
CE: cardioembolism
CI‐AKI: contrast‐induced AKI
CKD: chronic kidney disease
CNS: central nervous system
CRP: C‐reactive protein
CTA: computed tomography angiography
CysC: cystatin C
eGFR: estimated glomerular filtration rate
EMT: epithelial–mesenchymal transition
END: neurological deterioration
EPVS: enlarged perivascular spaces
ESRD: end‐stage renal disease
EVs: extracellular vesicles
EVT: endovascular therapy
GCs: glucocorticoids
GFAP: glial fibrillary acidic protein
GFR: glomerular filtration rate
HK‐2: human kidney 2
HT: hemorrhagic transformation
HTPA axis: the hypothalamic–pituitary–adrenal axis
ICH: intracerebral hemorrhage
ICUs: intensive care units
IGFBP7: insulin‐like growth factor‐binding protein 7
IL‐18: interleukin‐18
IL‐1β: interleukin‐1β
ILs: interleukins
IS: ischemic stroke
JAMs: junctional adhesion molecules
KDIGO: kidney disease: improving global outcomes
KIM‐1: kidney injury molecule 1
LAA: large‐artery atherosclerosis
MiRs: microRNAs
MMPs: matrix metalloproteinases
MRI: magnetic resonance imaging
mRS: modified Rankin scale
mt‐Nd6: mitochondrially encoded NADH dehydrogenase 6
NGAL: neutrophil gelatinase‐associated lipocalin
NK: natural killer
PA: pressure autoregulation
RAAS: renin–angiotensin–aldosterone system
RBF: renal blood flow
RBMEC: rat‐brain microvascular endothelial cells
ROS: reactive oxygen species
RVR: renal vascular resistance
SAH: subarachnoid hemorrhage
SAO: small artery occlusion
SCr: serum creatinine
SNGFR: single nephron glomerular filtration rate
SNS: sympathetic nervous system
SOE: stroke of other determined etiology
SUE: stroke of undetermined etiology
T2D: type 2 diabetes mellitus
TGF‐β: transforming growth factor beta
TIMP‐2: tissue inhibitor of metalloproteinases‐2
TJs: tight junction proteins
TNFs: tumor necrosis factors
TNF‐α: tumor necrosis factor alpha
TOAST: the trial of org 10172 in acute stroke treatment
Tregs: regulatory T cells
UACR: urine albumin‐to‐creatinine ratio
WML: white‐matter lesions
ZO: zonula occludens

## 1 Introduction

Stroke, a leading cause of mortality and disability worldwide, initiates a cascade of physiological responses extending beyond the central nervous system (CNS) [1]. Among the systemic consequences of stroke, renal dysfunction—manifesting as AKI and CKD—has emerged as a critical yet often‐overlooked area of concern. These conditions, while diverse in their clinical presentation, both represent pathophysiological states of the kidney that occur as a response to the brain–renal crosstalk initiated by a stroke.

AKI, characterized by a rapid decline in renal function, often marks the initial phase of kidney impairment following a stroke [2, 3]. If unresolved, this acute renal injury can progressively transition into CKD, a long‐term condition characterized by persistent kidney damage and a gradual loss of renal function over time [4].

This review seeks to elucidate the complex mechanisms underpinning the relationship between stroke and renal impairment, focusing on inflammatory factor release, blood–brain barrier (BBB) impairment, immune cell response, and the role of extracellular vesicles (EVs) and microRNAs (MiRs). By exploring these intertwined pathways, we hope to shed light on the systemic nature of stroke and its impact on renal health.

As we delve into the intricate dynamics of neuro–renal interactions following stroke, we also underscore the therapeutic implications of these findings, which could pave the way for novel strategies to mitigate stroke‐induced renal damage. The aim of this review is to investigate the brain–kidney axis between renal injury after stroke to provide more opportunities for early detection and potential therapeutic directions for patients in the clinical setting. At the same time, we acknowledge the gaps in our current understanding, emphasizing the need for further research in this fascinating intersection of neurology and nephrology.

## 2 Stroke and Renal Dysfunction: Clinical Insights

The cerebro–renal interaction is an emerging research focus due to its substantial implications for patient outcomes and overall healthcare management. In this section, we delve into the clinical dimensions of post‐stroke renal dysfunction, starting with the epidemiological evidence that underscores the frequency and severity of this often‐overlooked consequence of stroke.

### 2.1 Epidemiology of Kidney Injury Post‐Stroke

Stroke, a neurologically devastating disease, can concurrently instigate peripheral organ pathologies, including kidney dysfunction. Understanding this cerebro‐renal relationship is imperative given the ubiquitous nature of AKI and CKD—clinical conditions that typically lead to alterations in glomerular filtration rate (GFR), urine output, and relevant biomarkers [5, 6, 7].

**FIGURE 1:**
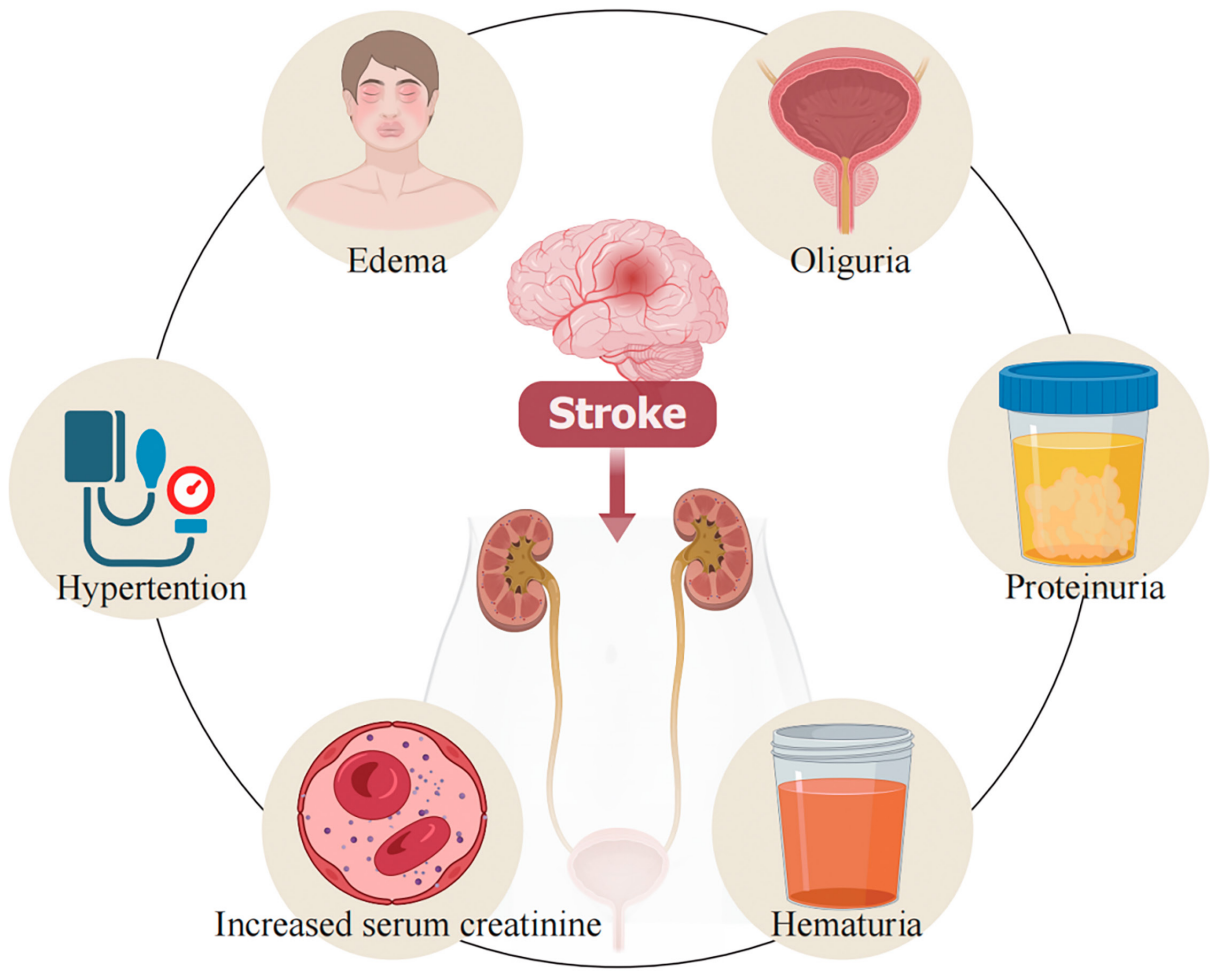
This chart lists relevant clinical manifestations and abnormal examination results that may be seen in patients with post‐stroke kidney injury.

However, the incidence of AKI and CKD following stroke is a contentious subject, with reported morbidity rates exhibiting substantial variation across clinical studies [8, 9, 10]. Meta‐analyses conducted in 2018 reported an incidence rate of 9.61% and 11.6% respectively, for AKI following stroke [8, 10]. Consequences of stroke‐induced renal insufficiency often include severe neurological sequelae and poorer patient outcomes, underscoring the clinical relevance of studying the brain‐kidney interplay in the context of stroke. Figure 1 lists relevant clinical manifestations and abnormal examination findings observed in patients with post‐stroke kidney injury.

### 2.2 Estimated Glomerular Filtration Rate (eGFR) as a Prognostic Indicator Post‐Stroke

Renal dysfunction, characterized by a persistent decline in renal function, can precipitate structural damage, severe clinical syndromes, accumulation of waste metabolites, and abnormal proteinuria discharge. In current clinical practice, the evaluation of renal function primarily relies on indicators like eGFR, albuminuria, and other related markers [11, 12, 13]. Progressive deterioration of these markers often heralds a poor prognosis in stroke patients [14, 15].

GFR is typically represented by surrogate markers such as serum creatinine (SCr) and urine output, serving as effective guides for clinical diagnosis. The eGFR is a critical diagnostic measure reflecting renal function. It quantifies the filtration capacity of the glomeruli, the kidneys' minute filtering units, assessing the volume of blood cleared of creatinine per minute. Frequently used to monitor and diagnose chronic kidney disease, eGFR considers variables like blood creatinine levels, age, gender, and body size [16]. Among these, creatinine‐based eGFR has amassed substantial clinical evidence, establishing itself as a reliable predictor of adverse outcomes post‐stroke, including recurrent stroke, cardiovascular events, stroke‐related disability, re‐hospitalization, and all‐cause mortality [17, 18, 19, 20].

A multicentric, randomized controlled trial studying acute intracerebral hemorrhage (ICH) patients from 21 countries demonstrated that lower eGFR values correlated with a higher risk of death or major disability at 90 days. However, there are also issues of generalizability arising from the clinical trial population [19]. According to the kidney disease: improving global outcomes (KDIGO) CKD Work Group, a decreased GFR is defined as GFR < 60 mL/min per 1.73 m^2^ (GFR categories G3a–G5) [6]. This renal dysfunction is associated with a higher incidence of new cardiovascular events, stroke‐related disability, recurrent stroke, and an increasing rate of hospital readmissions, thereby intensifying both short‐term and long‐term mortality [15, 21].

Interestingly, a number of studies suggest that, akin to low eGFR, a high eGFR could also indicate a poor prognosis in stroke patients, even after adjusting for confounding factors such as age, sex, and risk factors [7, 22, 23]. However, a prospective cohort study by Luo observed a differential association: higher eGFR was related to increased all‐cause mortality, while lower eGFR correlated with higher all‐cause mortality, stroke recurrence, and disability [23]. The strengths of this study include its large sample size of stroke patients from China. The limitation is the potential selection bias, as patients with missing baseline serum creatinine or lost to follow‐up within one year were not included in the study.

Notably, high eGFR might not reflect accurate renal function under conditions of substantial muscle mass loss, such as old age, amputations, paralysis, and chronic muscle disease [24]. Elevated eGFR can also manifest in early stages of diabetic nephropathy, and conditions like youth, type 2 diabetes mellitus (T2D), and heavy alcohol consumption are recognized predisposing factors for high eGFR. In contrast, low eGFR, but not high eGFR, is highly relevant to cerebral small‐vessel occlusive stroke in elderly patients [22]. Thus, patient comorbidities and other influencing factors could contribute to the observed eGFR changes post‐stroke. While renal insufficiency is frequently linked to poor stroke prognosis, current evidence is insufficient to support the utility of renal dysfunction in stroke risk stratification [25].

### 2.3 The Implications and Progression of AKI Following Stroke

As per the 2013 KDIGO Clinical Practice Guidelines, AKI is characterized as an abrupt deterioration in renal function, as indicated by an increased SCr (≥ 1.5 times the baseline value within 7 days or ≥ 26.5 μmol/L within 48 h), or diminished urine output (< 0.5 mL/kg/h for 6 h) [5, 26]. AKI is a clinical syndrome with a diverse etiology, resulting from direct renal damage and acute impairment of kidney function, and can be categorized into different stages based on SCr or urine output. Recent evidence posits cystatin C, interleukin‐18 (IL‐18), glutathione‐S‐transferase, insulin‐like growth factor‐binding protein 7 (IGFBP7), and tissue inhibitor of metalloproteinases‐2 (TIMP‐2) as potential biomarkers for early diagnosis of AKI via plasma or urine tests [13]. Current epidemiological evidence suggests that even transient, mild AKI can significantly impact the clinical outcomes of stroke, increasing disability and mortality rates [27, 28, 29].

Indicators related to renal function can directly signify the progression of AKI. A study in 2015 revealed a correlation between higher albumin–creatinine ratio (ACR) and lower eGFR with an elevated risk of AKI in patients with comorbidities like diabetes and hypertension [30]. In line with extant clinical studies, post‐stroke proteinuria has been associated with AKI, mirroring the relationship observed with eGFR. Retrospective analysis indicates that proteinuria exacerbates secondary AKI and increases one‐year mortality in stroke patients undergoing cerebral angiography. However, due to the low proportion of participants with proteinuria, this research was unable to analyze the dose‐effect relationship between proteinuria and outcomes [31]. After controlling for baseline comorbidities, it has been identified that baseline renal function is a potent independent predictor of short‐term survival post‐acute stroke and a risk factor for subsequent AKI [2]. Biomarkers indirectly linked with stroke have also been associated with AKI and poorer stroke prognosis [32]. Furthermore, a glycaemia‐based nomogram shows potential in predicting AKI in patients with ischemic stroke (IS) post‐endovascular therapy (post‐EVT) [33].

The medications or treatments administered to stroke patients post‐admission may only have minimal correlation with the incidence and severity of AKI [9, 19, 34]. Nevertheless, AKI might predominantly be triggered by the stroke itself, rather than the associated angiography and endovascular treatment post‐stroke. Studies suggest that neither thrombolytic therapy nor other related treatments for IS exacerbate AKI progression [34]. A clinical trial encompassing data from various specialized intensive care units (ICUs) from academic medical centers found a lower incidence of AKI, regardless of patients undergoing computed tomography angiography (CTA) or endovascular treatment [9]. Another study in 2016 illustrated that early intensive antihypertensive treatment for ICH positively impacted the incidence of 90‐day mortality or severe disability, irrespective of whether patients had a reduction in eGFR or not [19]. However, as renal insufficiency progresses, therapeutic efficacy diminishes, and it is significantly linked to adverse outcomes [9, 19, 35]. In a multivariate logistic regression analysis, researchers discovered that patients with albuminuria had a five‐fold higher risk of developing contrast‐induced AKI (CI‐AKI) compared to stroke patients without albuminuria [31]. In summary, the early detection and identification of AKI during the initial stages of stroke progression can serve as a crucial guide for improving the poor prognosis of stroke and mitigating the mortality associated with renal injury.

### 2.4 The Implications and Progression of CKD Following a Stroke

According to the KDIGO clinical practice guidelines, CKD is characterized by abnormalities of renal structure or function that persist for over 3 months, with a diagnostic threshold set at a GFR of less than 60 mL/min per 1.73 m^2^ and an ACR of 30 mg/g or higher [6]. CKD is a prevalent clinical condition with a higher incidence among high‐risk groups including the elderly, diabetics, and hypertensives. Notably, sensitive biomarkers of CKD do not entirely align with those of AKI. For instance, neutrophil gelatinase‐associated lipocalin (NGAL) and urinary kidney injury molecule 1 (KIM‐1), markers of renal tubular injury, are elevated in the context of CKD, but not AKI [11].

The brain and kidney share similar hemodynamic characteristics, including effective dynamic autoregulation capabilities. CKD and stroke can reciprocally influence each other's onset and progression by affecting hemodynamics and causing vascular structure disruptions. While many researchers are currently focusing on stroke incidence in CKD patients following vascular disease onset, the primary objective of this article is to explore the development of CKD in scenarios where stroke is the precipitating and leading cause, even long after the stroke occurrence.

Stroke patients often present with hypertension due to increased pressure, which further diminishes renal blood supply, exceeding the compensatory capacity of the kidneys. This can result in renal dysfunction manifestations such as reduced eGFR, proteinuria, and oliguria. Albuminuria, a marker of glomerular integrity, has been recognized in numerous studies as a potent clinical indicator for assessing the risk of adverse outcomes in patients with IS, particularly in predicting early neurological deterioration (END) and white‐matter lesions (WML) [31, 36, 37, 38].

The development of CKD can also forecast an unfavorable prognosis in IS patients, including recurrent stroke risks and heightened mortality [39, 40, 41]. In a multicenter cohort study involving patients with acute ischemic stroke (AIS) undergoing thrombolysis, CKD‐associated renal impairment was found to reduce thrombolysis efficacy without raising hemorrhagic complications, differing from AKI. This effect may primarily be attributed to the decrease in fibrinolysis and variance in infarct size [39]. Post‐stroke CKD signals an increase in localized brain damage, disturbances in the body's water and electrolyte balance, and in severe cases, end‐stage organ failure such as uremia.

## 3 Impact of Various Stroke Subtypes on Renal Function

Stroke is a complex disorder encompassing several subtypes, primarily including IS and intracerebral hemorrhage. In the trial of Org 10,172 in acute stroke treatment (TOAST) classification, IS is further categorized into five subtypes: large‐artery atherosclerosis (LAA), cardioembolism (CE), small artery occlusion (SAO), stroke of other determined etiology (SOE), and stroke of undetermined etiology (SUE) [42]. Patients may initially present with one stroke subtype and subsequently develop other subtypes. For instance, patients with IS may develop cerebral hemorrhage secondary to the natural progression of the disease or due to inappropriate treatment.

Each stroke subtype demonstrates unique clinical characteristics, with varied incidence and severity of accompanying renal injury. It has been reported that patients with ICH or aneurysmal subarachnoid hemorrhage (SAH) exhibit enhanced renal clearance. This elevated renal clearance can lead to subtherapeutic concentrations of renally eliminated drugs, resulting in decreased drug efficacy and potentially, medical complications [43].

Table 1 provides a summarization of renal dysfunction manifestations and related adverse prognosis associated with different stroke subtypes. However, it is crucial to note that current studies regarding each stroke subtype are not exhaustive. Thus, this table offers a partial snapshot of specific renal impairment manifestations and related prognostic outcomes for the various subtypes of stroke.

**TABLE 1:** Summary of the different kidney impairments and relevant prognosis associated with various stroke subtypes.

Stroke subtype	Kidney impairments	Relevant prognosis	References	Authors	Sample; mean age, year; follow‐up
Ischemic stroke	Albuminuria or high urine albumin‐to‐creatinine ratio (UACR)	END	[37]	Kanamaru et al. 2017	The stroke center at Nippon Medical School Hospital {*n* = 294}; 73.6 ± 11.9 years; No mention
		WML	[38]	Suda et al. 2017	The stroke center at Nippon Medical School Hospital {*n* = 284}; 72 years; No mention
	Rapid decline in eGFR	Cardiovascular events Recurrent stroke	[15]	Lee et al. 2013	The VISP trial {*n* = 3673}; ≥ 44.6 years; 2 years
	Increased SCr (the higher mean serum creatinine on admission)	High mortality	[7]	Luo et al. 2014	ACROSS{*n* = 4836}; ≥ 41 years; 1 year
	Increased serum urea, uric acid level	Lower odds of discharge	[14]	Husseinet al. 2017	The GWTG‐Stroke program {*n* = 232,236}; ≥ 65 years; No mention
	Decreased urine output	High rate of re‐hospitalization	[21]	Husseinet al. 2018	The GWTG‐Stroke program {*n* = 204,652}; ≥ 65 years; 1 year
		High at‐discharge death/disability	[18]	Nugroho et al. 2018	The Shiga Stroke Registry (SSR) {*n* = 2813}; ≥ 18 years; No mention
LAA	Low eGFR related to stroke severity	Poor functional outcome (modified Rankin Scale (mRS) > 3) Increased risk of 6‐month mortality	[44]	Yeh et al. 2015	The prospective Taiwan Stroke Registry {*n* = 8052}; ≥ 25.9 years; 6 months
	Reduction in eGFR	Increased risk of spontaneous HT (but not symptomatic HT)	[45]	Liu et al. 2016	The Chengdu Stroke Registry {*n* = 1645}; 62.9 years; No mention
CE	Renal insufficiency		[46]	Kudo et al. 2012	Yamagata City Hospital, Saiseikan, Japan {*n* = 525}; 74.1 ± 12.1 years; 1 year
SAO	Albuminuria Decline in eGFR	END Increasing enlarged perivascular spaces (EPVS) severity Cerebral microbleeds progression	[36]	Umemura et al. 2014	The stroke center in Chubu Rosai Hospital {*n* = 85}; 69.8 years; No mention
	High serum CysC level	Increased total MRI burden	[47]	Yang et al. 2017	The Department of Neurology at Third Affiliated Hospital of Soochow University {*n* = 210}; 67.17 ± 12.35 years; No mention
SOE					
SUE	Advanced renal dysfunction	All‐cause mortality Poor functional outcome at third‐month and sixth‐month post‐stroke	[48]	Chua et al. 2021	Taiwan Stroke Registry {*n* = 3775}; 66.6 ± 14.5 years; 1 year
Hemorrhagic stroke					
ICH	Reduced eGFR	Increased death/disability	[35]	Fukuda‐Doi et al. 2021	ATACH‐2 {*n* = 974}; ≥ 47 years; 90 days
	Combined with CKD	Slightly worse care Substantially higher mortality associated with CKD	[40]	Ovbiagele et al. 2014	The GWTG‐Stroke program {*n* = 5,113,059}; (Patient with CKD) 72.5 years; (Patient without CKD) 67.3 years; No mention
	The higher mean SCr on admission		[49]	Snarska et al. 2016	The Department of Neurology, Medical University Hospital Bialystok {*n* = 129}; 64.80 ± 15.76 years; No mention
	Proteinuria		[46]	Kudo et al. 2012	Yamagata City Hospital, Saiseikan, Japan {*n* = 525}; 74.1 ± 12.1 years; 1 year
SAH	Enhanced renal clearance		[43]	Morbitzer et al. 2019	Neurosciences ICU in a tertiary care academic medical center {*n* = 50 + 30 = 80}; 57.2 ± 10.7 years; No mention

## 4 Is There a Link Between AKI and CKD Following Stroke?

AKI is a clinical condition marked by rapid deterioration of renal function, which is often accompanied by hypovolemia, sepsis, and nephrotoxic drugs. Various vasoconstrictive substances, including catecholamines, are released due to the activation of the sympathetic system following an acute stroke, leading to a systemic redistribution of blood flow. This prioritizes blood supply to the heart and brain, but significantly diminishes renal blood flow, thereby inducing a state of renal ischemia.

On the other hand, CKD, a chronic or progressive renal disorder, is characterized by inflammation‐induced myofibroblast transformation, proliferation, capillary disintegration, and thinning [4]. The stroke often elevates plasma levels of interleukin‐1 beta (IL‐1β). When a stroke occurs, transforming growth factor beta (TGF‐β) overexpression can activate downstream Smad signaling, contributing to renal fibrosis [50, 51, 52]. Additionally, stroke often results in upregulated expression of C‐reactive protein (CRP) and reactive oxygen species (ROS), which mediate renal tissue fibrosis through a TGF‐β1‐dependent mechanism, indirectly upregulating the downstream Smad3 signaling pathway [53].

**FIGURE 2:**
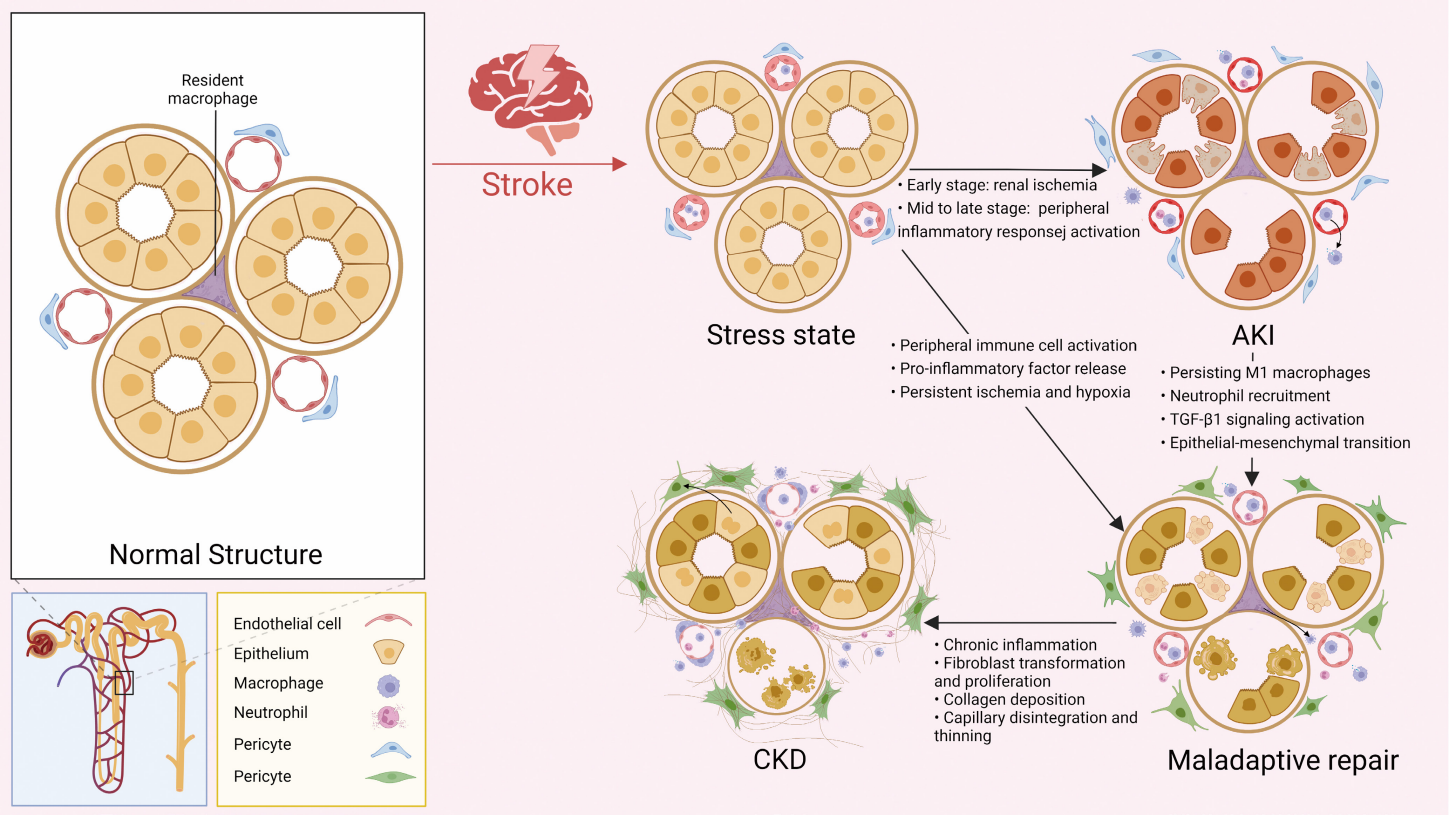
Schematic of the pathologic process of AKI and CKD after stroke. This diagram illustrates the changes that occur in renal tubular tissue following a stroke, leading to AKI and CKD. Initially, the kidneys experience ischemia and hypoxia due to high vascular resistance, which can result in acute tubular cell necrosis and endothelial cell damage, causing AKI. In the mid to late phase, brain macrophages are activated and released into the blood, further causing activation of peripheral immune cells (e.g., renal resident macrophages) as well as neutrophil recruitment. Large numbers of leukocytes infiltrate the kidney, M1 phenotype macrophages are continuously expressed, and maladaptive repair of the renal tubules occurs. Stimulated by pro‐inflammatory and other injury factors, pericytes separate from the endothelium, resulting in capillary thinning. Additionally, epithelial or endothelial cells may undergo epithelial‐mesenchymal transition (EMT), transforming into mesenchymal cells through TGF‐β1 signal pathway activation and overexpression of MiRs. Pericytes proliferate and differentiate to produce myofibroblasts, which promote collagen deposition in the kidney and cause renal fibrosis, ultimately leading to and the development of CKD.

Epidemiological studies highlight the complex interplay between AKI and CKD in stroke patients. Patients who suffer from AKI are at a higher risk of developing CKD, but differentiating the individual contributions of AKI and shared risk factors such as diabetes, hypertension, proteinuria, and coronary artery disease to CKD is a challenge [54]. AKI‐induced changes such as myofibroblast transformation, proliferation, fibrosis, and vascular disintegration are often implicated in the development of CKD [3, 43]. Figure 2 illustrates the potential pathological processes of AKI and CKD following a stroke. Moreover, AKI's indirect contribution to CKD progression, by influencing hemodynamic disturbances and renal load in stroke patients, is noteworthy.

In conclusion, stroke‐associated AKI doesn't directly lead to CKD. AKI is more as an acute pathophysiology of the kidneys which is related to the progression of peripheral neurological and circulatory conditions following stroke. Instead, it is related to maladaptive repair of abnormal or necrotic renal tubules due to repeated stimulation, caused by underlying diseases such as high vascular resistance, coronary artery disease, and diabetes mellitus [4].

## 5 Mechanisms Underlying Renal Impairment Post‐Stroke: Hemodynamic and Neuroendocrine Pathways

**FIGURE 3:**
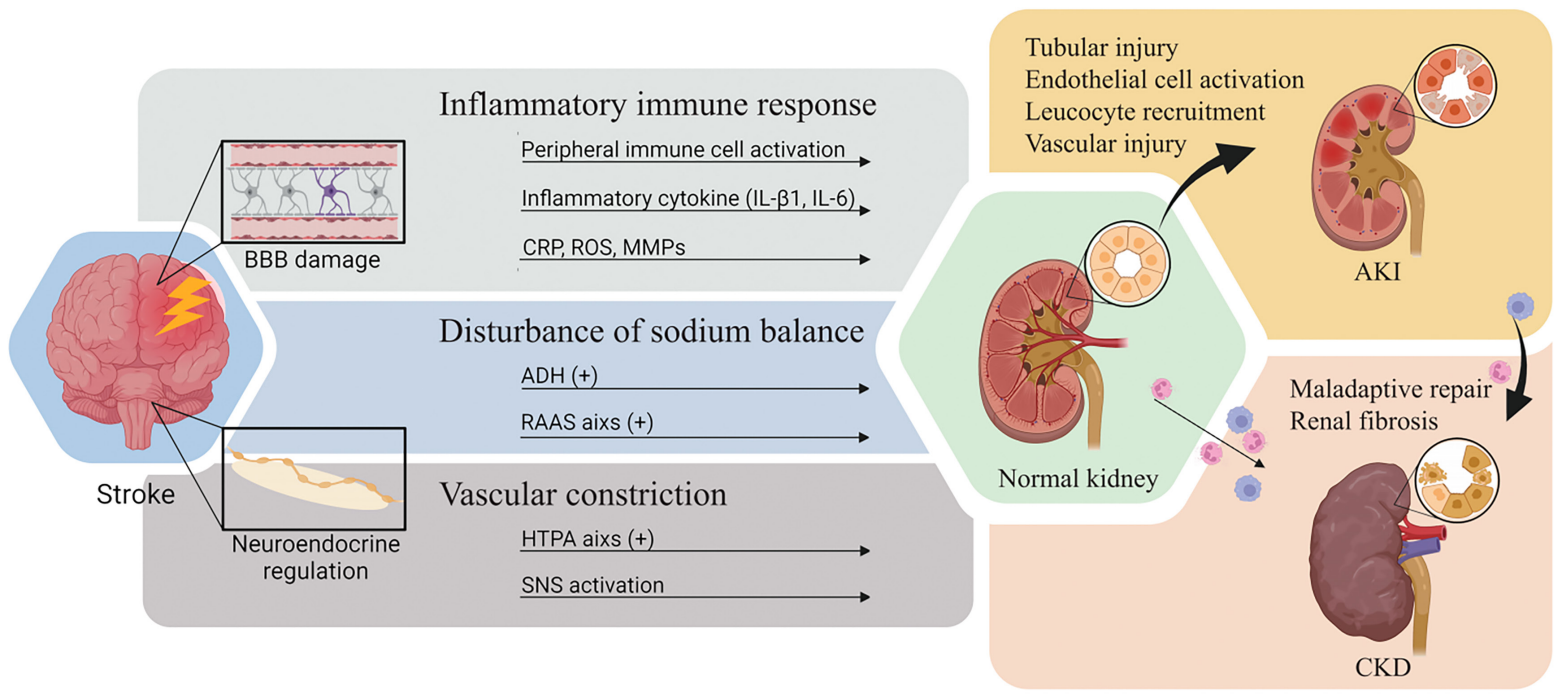
Schematic overview of the mechanisms and pathways involved in renal impairment after stroke. Renal impairment after stroke involves two major pathways: The inflammatory immune pathway and the neurohumoral pathway. In the inflammatory immune pathway, the BBB is compromised after stroke, leading to the release of pro‐inflammatory substances from the brain into the blood and the activation of peripheral inflammatory immune responses. The neurohumoral pathway involves the activation of the RAAS axis, the HTPA axis and the SNS. Multiple vasoconstrictor substances and related hormones act on receptor targets, resulting in hemodynamic disturbances and an acute ischemic state in the kidney. Multiple mechanisms promote the development of AKI or CKD in the kidney, while stroke as an acute injury promotes the progression of AKI to CKD.

The kidneys and brain sustain homeostasis through a complex interplay via neuroendocrine pathways [55]. A stroke, however, may disturb this balance by overstimulating the CNS and sympathetic nervous system (SNS) and impairing the BBB. This allows inflammatory mediators and immune‐related substances from the brain to enter the bloodstream, inciting systemic circulatory inflammatory immune responses that indirectly affect the kidney. Stroke‐related renal failure is believed to arise from the combined effects of these neuroendocrine and inflammatory‐immune pathways, along with other pathophysiological mechanisms [56, 57, 58]. Figure 3 depicts the potential mechanisms and pathways underlying renal dysfunction following a stroke. This section explores these mechanisms in‐depth.

### 5.1 Hemodynamic Mechanisms and Autoregulation

Despite serving different roles, the brain and kidneys share a key feature: robust pressure autoregulation (PA), which maintains stable blood flow despite variations in perfusion or blood pressure (BP) levels [59, 60]. Cerebral vasculature maintains constant flow even when arterial BP varies between 50 and 150 mmHg, while renal autoregulation maintains a steady renal blood flow with BP ranges of 80–180 mmHg [61]. Factors such as age, gender, comorbidities, anesthetic drugs, and vasoactive agents can influence these autoregulatory boundaries [62].

Post‐stroke cerebral autoregulation impairments can lead to hypoperfusion, altered microvascular pressure, BBB integrity loss, and microbleeds [63, 64]. A retrospective study of dynamic nomogram for predicting acute kidney injury in patients with acute ischemic stroke has indicated that compromised cerebral autoregulation significantly correlates with renal dysfunction during the acute phase (< 6 h) of AIS. But single‐center population selection bias and the constant revision of treatment protocols may lead to decreased predictive performance [64]. This disturbance in cerebral blood flow post‐stroke indirectly contributes to renal hemodynamic alterations.

### 5.2 Neuroendocrine Pathway Over‐Activation

#### 5.2.1 The Hypothalamic–Pituitary–Adrenal (HPA) Axis

The HPA axis is a servocontrol system involving direct actions and feedback interactions [65]. Post‐stroke, increased blood cortisol levels are observed, correlating with injury severity and location [66, 67, 68, 69]. The primary cortisol hormones—glucocorticoids (GCs), mineralocorticoids, and sex hormones—can impact renal function following a stroke. Specifically, GCs can affect glomerular function, leading to a short‐term increase in GFR and proteinuria [70, 71]. High cortisol levels correspond with increased renal vascular resistance (RVR) and renal blood flow (RBF), affecting single nephron glomerular filtration rate (SNGFR) and consequently increasing systemic GFR [72].

#### 5.2.2 The Renin‐Angiotensin‐Aldosterone System (RAAS)

The RAAS rapidly activates post‐stroke, resulting in the upregulation of RAAS‐related substances, with angiotensin II playing a crucial role [73]. High levels of aldosterone have been associated with poor stroke outcomes [74]. Additionally, angiotensin II is known to induce pro‐inflammatory factor IL‐6 production, while aldosterone stimulates ROS production, increasing endothelial cell permeability and promoting renal fibrosis [75, 76].

### 5.3 The Activation of SNS

AIS triggers a stress response, leading to SNS activation and increased release of catecholamines such as epinephrine and norepinephrine [77]. This provokes renal vasculature constriction, leading to pre‐renal insufficiency [78, 79], and potentially causing acute kidney injury due to prolonged renal ischemia and hypoxia [4]. Hence, the SNS plays a significant role in the early phase of stroke, despite its detrimental impact on renal hemodynamics.

In summary, post‐stroke renal impairment arises from complex, interwoven neuroendocrine and hemodynamic pathways. A comprehensive understanding of these mechanisms will provide valuable insight into effective therapeutic strategies to minimize renal damage following stroke.

## 6 Mechanisms of Renal Impairment Post‐Stroke: Inflammatory Mediators and Immune Response

The breakdown of the BBB, neuroendocrine activation, and the systemic dispersion of brain‐derived inflammatory‐immune substances can instigate a peripheral sterile inflammatory response [80, 81]. Stroke‐induced sterile inflammation in the brain triggers a multifaceted immune response, involving an array of immune cells, including microglia/macrophages, neutrophils, astrocytes, and T cells [82]. These cells orchestrate a large‐scale release of inflammatory mediators such as interleukins (ILs), tumor necrosis factors (TNFs), and chemokines [57, 83]. These processes lead to the widespread distribution of inflammatory factors and immune cells, with the kidneys being particularly vulnerable to their effects. Prolonged exposure to inflammatory stimuli can provoke a series of renal structural changes, including myofibroblast accumulation and collagen deposition, culminating in renal fibrosis [84, 85].

Inflammatory pathways stimulate renal fibrosis after stroke. Therefore, inhibiting inflammation becomes an effective therapeutic strategy to alleviate renal disease after stroke. Such a strategy underscores the necessity of understanding the cascading effects of stroke‐induced inflammation on kidney health and function.

### 6.1 BBB Impairment in Post‐Stroke Renal Injury

The BBB is a critical biochemical barrier that sustains CNS homeostasis. It controls the passage of essential nutrients and metabolic waste products via the expression of a variety of ion transport proteins and channels [80, 86]. The BBB is composed of tight junctions between brain microvascular endothelial cells that regulate paracellular diffusion between adjacent endothelial cells. The transmembrane tight junction proteins (TJs) encompass claudins, occludin, tricellulin, junctional adhesion molecules (JAMs), and accessory proteins such as zonula occludens (ZO) proteins [87].

BBB damage is a cardinal pathological characteristic of stroke that typically commences early (usually within 6 h post‐stroke) and persists [88]. The increased permeability of the BBB during a stroke permits easier entry of solutes into the circulation, contributing to the development of vasogenic edema and hemorrhagic transformation (HT) [89, 90]. Initial BBB damage in AIS primarily stems from hypoxia and nutrient deprivation brought about by persistent post‐stroke hypoperfusion. This stressor stimulates an increase in the expression of matrix metalloproteinases (MMPs) and ROS in endothelial cells and infiltrating neutrophils, leading to the aberrant expression of linker proteins. Subsequent neuroinflammation contributes to irreversible BBB damage [80, 81].

BBB damage is a necessary precursor to secondary renal damage following stroke. The early translocation of pro‐inflammatory factors, immune‐related substances, and upstream regulatory signals of pathways from the brain injury site into the circulation via the BBB may be a critical step in the evolution of AKI. Further, functional BBB deficiency and the upregulated expression of hormones, such as antidiuretic hormone (ADH) and aldosterone, give rise to systemic circulating ion disturbances that exacerbate renal insufficiency. A deeper understanding of BBB damage mechanisms and the potential for transporting various types of regulatory factors into the circulation to reach the kidney will aid researchers in further exploring the signal pathways and mechanisms implicated in renal damage at different stages of stroke. Consequently, safeguarding the BBB and reducing its permeability during stroke onset might represent a potential therapeutic strategy to mitigate renal impairment post‐stroke.

### 6.2 Interplay of Immune Cells in Stroke‐Induced Brain and Kidney Injury

Stroke elicits a complex immune response that involves a broad array of immune cells. Among these, monocytes/macrophages, neutrophils, natural killer (NK) cells, and T cells are notable for their substantial impact on both stroke‐induced brain injury and subsequent peripheral organ damage, particularly in the kidneys.

#### 6.2.1 Monocytes/Macrophages

The response to IS is rapidly initiated by the activation of resident immune cells in the CNS, the monocytes/macrophages [91]. Within hours of the onset of IS, numbers of microglia in the border zone of the ischemic area increases rapidly which are induced to release a plethora of cytotoxic components, contributing to an inflammatory environment that exacerbates neuronal damage [92]. This inflammatory milieu is not confined to the CNS but permeates the systemic circulation, as these activated monocytes/macrophages translocate into peripheral tissues. In the kidney, their ongoing pro‐inflammatory secretions contribute to renal dysfunction, fostering the development of fibrosis, a chronic kidney condition characterized by an excess accumulation of fibrous connective tissue. Therefore, regulation of monocyte/macrophage activation and response post‐stroke can potentially offer a therapeutic avenue to mitigate deleterious effects on both brain and kidney function.

#### 6.2.2 Neutrophils

Neutrophils, the most abundant white blood cells, are another critical player in the immune response to stroke. Following a stroke, neutrophils are quickly mobilized to the brain, where they intensify the inflammatory response through the release of pro‐inflammatory cytokines, chemokines, and ROS [93]. Beyond their damaging role in the CNS, these activated neutrophils migrate into the systemic circulation, instigating widespread inflammation that impacts peripheral organs, including the kidneys. In the renal tissue, neutrophils potentiate injury by releasing harmful substances and amplifying the inflammatory response, underscoring the necessity of strategies to moderate neutrophil activation to alleviate stroke‐induced renal damage.

#### 6.2.3 NK Cell

NK cells, the immune system's initial defenders against infections and diseases, are activated and recruited to the brain following stroke. Their contribution amplifies the inflammatory environment in the brain, leading to more extensive damage [94]. Systemically, the stroke‐induced activation of NK cells disrupts immune homeostasis, which potentially heightens the susceptibility of peripheral organs, such as the kidneys, to inflammatory insult.

#### 6.2.4 T Cell

T cells play a nuanced role in stroke. They can perpetuate the post‐stroke inflammatory response, contributing to exacerbated brain damage. Nevertheless, certain subtypes, specifically regulatory T cells (Tregs), can restrain the immune response and limit tissue damage [95]. Systemically, these activated T cells traverse the body and localize in various organs, including the kidneys, where their impact diverges based on their subtype. Effector T cells can provoke inflammation and instigate AKI, while Tregs may mitigate renal injury through suppression of the inflammatory response.

In summary, understanding the complex interplay of these immune cells in stroke‐induced brain and subsequent renal damage is paramount. This knowledge could pave the way for the development of therapeutic interventions targeting these cells to alleviate both the primary brain injury and secondary renal damage ensuing stroke.

### 6.3 Systemic Inflammatory Response and Oxidative Stress Post‐Stroke: The Role of pro‐Inflammatory Factors

Stroke instigates a series of biochemical responses, prominently featuring the production and release of pro‐inflammatory factors and oxidative stress products from microglia, neutrophils, and endothelial cells in ischemic brain tissue. Subsequent to the disruption of the BBB, these cells expel cytokines (e.g., ILs, tumor necrosis factor alpha (TNF‐α), chemokines), and brain‐derived antigens (e.g., enolase, S100b, and glial fibrillary acidic protein (GFAP)) into the bloodstream [83, 96]. These bioactive substances can stimulate both distant organs and the peripheral immune system. Evidence of brain‐derived antigens and specific antibodies can be detected in the tonsils and lymph nodes of stroke patients [97, 98].

In the context of stroke, particularly as observed in animal models, systemic immune responses are noticeably altered. Post‐stroke, an observable increase in the presence of various peripheral immune cells, including lymphocytes, monocytes, and neutrophils, is seen in the circulatory system. This increase is often paired with anatomical changes in immune organs, such as the reduction of spleen volume. These peripheral immune cell responses underscore the systemic nature of immune activation following a stroke [90].

The activation of these immune cells results in the production of an array of pro‐inflammatory factors and oxidative stress products. The subsequent release of these substances into the bloodstream triggers a systemic inflammatory immune response, contributing to secondary injury. The implications of these immune responses post‐stroke suggest a significant interplay between the CNS and the peripheral immune system. Understanding this interaction is crucial in exploring the mechanisms behind the secondary effects of stroke on peripheral organs and potentially designing therapeutic interventions to mitigate these effects. Several substances integral to the inflammatory immune response post‐stroke are discussed in detail below.

#### 6.3.1 ILs

ILs can be broadly categorized into anti‐inflammatory and pro‐inflammatory classes. Notably, IL‐6 demonstrates variable roles in different phases of stroke—acute, subacute, and long‐term [99]. Serum concentrations of IL‐6 are significantly elevated after stroke onset [100]. IL‐6 exhibits pleiotropic effects, playing a pro‐inflammatory role in the development of AKI in the short‐term, and contributing to renal fibrosis in the long term by promoting the upregulation of multiple fibrotic genes in the kidney, including α2‐procollagen, TGF‐β, and plasminogen activator inhibitor‐1 [101, 102, 103].

#### 6.3.2 CRP

CRP, an acute‐phase protein and immune mediator activated by complement, can permeate the BBB [104, 105]. Observations by Pecoits‐Filho et al. and Ladenvall et al. show elevated plasma CRP levels early in IS, and this condition also occurs after CKD and renal failure [52, 106].

CRP mediates the renal inflammatory response via a CD32‐NF‐κB‐dependent mechanism. Inflammatory signaling‐stimulated CRP notably upregulates pro‐inflammatory cytokines/chemokines (such as TNF‐α, IL‐1β), drives macrophage polarization toward the M1 phenotype, inhibits conversion to the M2 phenotype, and increases renal infiltration of F4/80+ macrophages through an NF‐κB‐dependent mechanism, triggering severe renal inflammation [107, 108, 109, 110]. Therefore, understanding these pro‐inflammatory factors and oxidative stress products is crucial in developing therapeutic strategies for stroke and its associated renal complications.

#### 6.3.3 ROS

ROS broadly refer to oxygen‐derived free radicals and non‐free radicals possessing potent chemical reactivity [111]. In vitro, ROS instigate caspase‐3‐mediated damage to TJs, leading to increased permeability in rat brain microvascular endothelial cells (RBMEC) [112]. This phenomenon is associated with alterations in both the BBB and the renal filtration barrier.

As a continuation from the previous discussion on the role of pro‐inflammatory factors and oxidative stress products in stroke‐induced renal impairment, another critical aspect to consider is the contribution of EVs and MiRs in this pathological process. Their involvement is particularly noteworthy as they represent complex intercellular communication systems that influence disease progression and, hence, potential therapeutic targets. The specifics will be explained in the next section.

## 7 EVs and MiRs: Mediators of Post‐Stroke Renal Fibrosis and CKD

EVs and MiRs are hypothesized as crucial effectors in the progression of renal fibrosis and CKD following stroke [55]. EVs, lipid bilayer membrane structures, function as cellular messengers through the transport of various biomolecules like lipids, proteins, and nucleic acids. These biomolecules modulate the interaction between parental and recipient cells, subsequently influencing the recipient cell function [113]. In the context of fibrotic diseases, EVs serve as critical conduits of protofibrillar signaling, mediating the continuous deposition of diverse insoluble interstitial collagens [114].

MiRs are small, non‐coding RNA molecules with potent regulatory effects. Abnormal secretion or repression of numerous miRs has been linked to the progression of renal fibrosis. Stroke can induce alterations in miRs expression, with aberrant up inflammatory‐immune and downregulation of several miRs observed in the sera of young stroke patients [115].

MiR‐21, for example, facilitates renal fibrosis by enhancing TGF‐β signaling [116]. Additionally, in renal tubular epithelial cells from patients with renal fibrosis, an increased expression of miR‐34a is observed. MiR‐34a binds to and downregulates the expression of Klotho, an endogenous inhibitor of renal fibrosis. This overexpression of miR‐34a, induced by TGF‐β1, promotes the EMT in human kidney 2 (HK‐2) cells [117].

Several models of CKD, including those induced by obstruction, ischemia/reperfusion, mitochondrial miRs, and albumin overload, have demonstrated an upregulated expression of miR‐214. In vitro, miR‐214 disrupts mitochondrial oxidative phosphorylation in CKD by binding to specific targets, namely the mitochondrial genes mitochondrially encoded NADH dehydrogenase 6 (mt‐Nd6) and mt‐Nd4l [118]. Furthermore, miR‐184 and microRNA‐122‐5p have also been implicated in renal fibrosis [119, 120].

In conclusion, the regulation of EVs and aberrantly up‐ or down‐regulated miRs represent promising therapeutic targets for mitigating the progression of CKD following stroke.

## 8 Current Limitations and Future Research Directions

Our understanding of the interplay between stroke and renal impairment has substantially advanced over recent years, but several challenges and research opportunities remain, mainly centered around deciphering complex mechanisms, improving diagnostic tools, and developing effective therapies.

Firstly, we emphasize the potential benefit of early detection or prevention of brain‐kidney axis‐related complications, which may improve patient prognosis. We suggest that clinicians may include monitoring of kidney‐related markers such as blood creatinine and proteinuria as one of the important things in stroke care. The quest for improved diagnostic and prognostic tools also persists. Future research can focus on the identification of novel biomarkers that provide enhanced accuracy and reliability, potentially using high‐throughput omics technologies to discover novel disease markers.

Simultaneously, targeting the specific mechanisms of the neuroendocrine pathway and inflammatory response, we can investigate the relevance this may include interventions such as neuroprotective agents that simultaneously benefit renal function, or attenuating the systemic inflammatory response to inhibit the interaction between the two systems.

As well, while current studies have shed light on various immune cells, cytokines, and inflammatory mediators in driving stroke‐induced renal damage, gaps in our understanding persist. Specifically, more detailed explorations are needed on the exact mechanisms of fibrosis and extracellular matrix accumulation. Future research should leverage advanced techniques such as single‐cell RNA sequencing and proteomics to elucidate the role of different cell types and biomolecules involved in renal impairment post‐stroke.

Next, the intricate role of the autonomic nervous system, specifically the sympathetic and parasympathetic branches, and endocrine pathways in mediating the stroke's effects on kidney function, present another complex area of study. Although previous research has addressed these pathways, the dynamic and multifaceted nature of these interactions requires further examination, ideally through integrative methodologies, encompassing multi‐omics analyses, and state‐of‐the‐art imaging techniques.

The challenge of translating findings from animal models to human patients is another area where research can focus. The complexity of human disease demands a higher emphasis on translational studies, bridging the gap between animal models and clinical applications. This could involve the use of human‐derived organoid models, which can better replicate human physiological conditions.

## 9 Summary and Conclusion

In summary, the intricate relationship between stroke and renal impairment is an area of biomedical research that has gained considerable attention due to its profound implications for patient outcomes and management. The post‐stroke pathophysiological cascade involving immune cell activation, pro‐inflammatory factor release, oxidative stress, and the involvement of signaling molecules and EVs all contribute to renal impairment. This complex interplay underscores the multifaceted nature of stroke‐induced renal damage.

This review summarizes the existing clinical studies on post‐stroke kidney injury and integrates and screens the different literature for the same indicators and trends presented. Therefore, the review is mainly a selection of representative study results for presentation, which may be subject to potential biases such as selection bias and publication bias. Some of the literature with different trends from the mainstream findings were interpreted and analyzed in more detail, but some omissions cannot be ruled out.

Current research has significantly advanced our understanding of these interactions, identifying key mechanisms and highlighting the importance of the BBB and immune responses. However, numerous questions and challenges remain. These include the need for deeper mechanistic insights, advanced diagnostic and prognostic tools, translational studies to bridge the gap between animal models and human patients, and effective therapeutic interventions.

In conclusion, further research is necessary to unravel this complex interplay, leading to improved patient care strategies. By focusing on the highlighted challenges and utilizing innovative approaches, we can work toward a future with better management and therapeutic strategies for stroke patients at risk of renal impairment.

## Ethics Statement

The authors have nothing to report.

## Consent

The authors have nothing to report.

## Conflicts of Interest

The authors declare no conflicts of interest.

## Data Availability

Authors confirm that all relevant data are included in the article.
